# Readiness for Discharge from Hospital after Myocardial Infarction: A Cross-Sectional Study

**DOI:** 10.3390/ijerph18136937

**Published:** 2021-06-28

**Authors:** Paulina Hydzik, Ewelina Kolarczyk, Wojciech Kustrzycki, Grzegorz Kubielas, Marta Kałużna-Oleksy, Remigiusz Szczepanowski, Bartosz Uchmanowicz

**Affiliations:** 1 Department of Clinical Nursing, Faculty of Health Sciences, Wroclaw Medical University, 51-618 Wroclaw, Poland; paulina.hydzik@umed.wroc.pl (P.H.); wojciech.kustrzycki@umed.wroc.pl (W.K.); grzegorz.kubielas@umed.wroc.pl (G.K.); bartosz.uchmanowicz@umed.wroc.pl (B.U.); 2 Department of Gerontology and Geriatric Nursing, Faculty of Health Sciences in Katowice, Medical University of Silesia, 40-752 Katowice, Poland; ewelinakol@o2.pl; 3 Polish National Health Fund (NFZ), Department of Health Care Services, Central Office in Warsaw, 02-528 Warsaw, Poland; 4 1st Department of Cardiology, University of Medical Sciences in Poznan, Lord’s Transfiguration Clinical Hospital in Poznan, 61-848 Poznan, Poland; marta.kaluzna@wp.pl; 5 Department of Public Health, Faculty of Health Sciences, Wroclaw Medical University, 51-618 Wroclaw, Poland

**Keywords:** myocardial infarction, readiness for hospital discharge, acceptance of illness, health education, prevention

## Abstract

Myocardial infarction (MI) is a common cause of cardiovascular deaths. Education of patients with myocardial infarctions essential to prevent further cardiovascular events and reduce the risk of mortality. The study aimed to evaluate the associations between patients’ readiness for hospital discharge after myocardial infarction, acceptance of illness, social, demographic, and clinical factors. The study used a cross-sectional design and included 102 patients, who were hospitalized for myocardial infarction after percutaneous coronary intervention (PCI). Two questionnaires were used: The Readiness for Hospital Discharge After Myocardial Infarction Scale (RHD-MIS) and Acceptance of Illness Scale (AIS). Low readiness characterized nearly half of patients (47.06%), 27.45% of patients showed an intermediate level of readiness, while 25.49% of patients had high readiness. Readiness for hospital discharge was higher among younger patients, respondents living in relationships, living with a family, with tertiary or secondary education, and professionally active. Acceptance of illness was higher among male patients, respondents living in relationships, and family, with secondary education and professionally active. The AIS score positively correlated with readiness for hospital discharge.

## 1. Introduction

Despite the trend observed in the last 30 years towards reducing mortality from ischemic heart disease, myocardial infarction is one of the most common causes of death globally [1,2]. It is estimated that about 20% of all deaths in Europe are caused by ischaemic heart disease, with significant differences between European countries. It causes close to half of all deaths in Europe, with the proportion of total deaths attributable to heart diseases in Europe greater for women (51%) than men (42%) [3,4]. According to the Central Statistical Office, cardiovascular diseases (CVD) account for nearly 50% of all deaths in Poland [5]. In 2013, myocardial infarction (MI) caused about 15 thousand deaths [6,7]. 

The correlation between risk factors and MI is driven by known variables: cigarette smoking, lipid concentrations, hypertension, diabetes, obesity, diet, physical activity, alcohol use, and psychological factors [8]. The most important role plays smoking, lipid abnormalities, hypertension, and diabetes, representing significant risk factors for MI in younger patients than in older subjects [9]. On the other hand, the perspective of changes from high-risk to low-risk behaviors including healthy diet (e.g., Mediterranean diet) and moderate alcohol consumption reduces the incidence of MI [10]. Myocardial infarction in adults below 45 years of age accounts for approximately 10% of all cases. It affects mostly men, but the proportion of women is steadily increasing. Additionally, the different mechanisms that lead to MI and risk factors may be observed in younger and older individuals. Because most risk factors are at least partially modifiable, it is believed that most cases of MI at a young age can be prevented [11]. 

Readiness for hospital discharge refers to the ability of patients to cope in the community after transitioning from an acute care hospital [12]. Studies on readiness for discharge have shown that it affects patient readmission [13]. During hospitalization, the measures carrying out secondary prevention can be implemented effectively, although they are limited in time. The patient should strive to change their lifestyle and eliminate the risk factors that may lead to another MI. Given this view, the patients’ knowledge about the disease should be assessed from their perspective, which will enable a comprehensive insight into the medical care system of the patient after myocardial infarction [14]. 

Treatment of patients after a cardiac incident does not end in the hospital unit. Cardiological rehabilitation, appropriate education, and planning of optimal therapy, and assistance in complying with behavioral recommendations lead to reduced future events [15]. Preparation of the patient for independent functioning after the myocardial infraction allows for a return to daily life and increases acceptance of illness [15,16].

Although the assessment of patient’s readiness for hospital discharge has been identified as an important component of clinical practice, there is still little research on the other factors contributing to increasing readiness to discharge in cardiac patients. Thus, the study aimed to assess associations between patients’ readiness to be discharged after myocardial infarction, acceptance of illness, and social, demographic, and clinical factors.

## 2. Materials and Methods 

### 2.1. Study Design, Settings, and Participants

The study used a cross-sectional survey on a selected group of patients (*n* = 102) of the Cardiological Rehabilitation Unit of the John Paul II Vratislavia Medical Hospital in Wrocław, hospitalized due to myocardial infarction within the period of February and September 2019. The inclusion criteria for the study were adult participants diagnosed with myocardial infarction, as well STEMI as NSTEMI treated with PCI (Percutaneous Coronary Intervention) [17,18]. 

### 2.2. Research Instruments

The research was carried out using two standardized measures: The Readiness for Hospital Discharge After Myocardial Infarction Scale (RHD-MIS) [19] and the Acceptance of Illness Scale (AIS) [20].

The RHD-MIS scale evaluates readiness for discharge of patients after myocardial infarction [19]. The questionnaire includes three subscales: (1) subjective assessment of patient knowledge about the disease, (2) objective assessment of patient knowledge about the disease, and (3) patient expectations. For the measurements of readiness, the centile standards were constructed to express low, intermediate, and high values. A score from 0 to 3 was assigned for each RHD-MIS item. A patient who scores above 57 points has high readiness, with scores less than 44 points has low readiness, while the range from 44 to 57 points indicates a patient with an intermediate level of readiness. RHD-MIS is considered a reliable and relevant tool for measuring patient readiness for discharge [19].

The Acceptance of Illness Scale (AIS) was used to quantify the factor of acceptance of illness. High AIS scores indicate better adaptation and less dependency of the adult patient. The scale contains eight statements that describe the consequences of poor health. The measure of acceptance of illness is the sum collected from the subscales: 8–18 points indicate low acceptance, 19–29 points indicate the intermediate level, and the range of 30–40 points indicates acceptance of the health situation at a satisfactory level [20].

Moreover, the study collected demographic and epidemiological information, which included queries about gender, age, marital status, place of residence, education, professional activity, comorbidities, type of myocardial infarction, type of pharmacological treatment undertaken, smoking as well as hospitalization and rehabilitation lengths of stay.

### 2.3. Statistical Analyses

The analysis of quantitative variables used the mean, standard deviation, median, quartile, minimum and maximum. For the qualitative variables, frequencies and percentages were calculated. The differences between the two groups were examined using the Mann-Whitney test. Comparisons between three and more groups were performed with the Kruskal-Wallis test. Post-hoc tests for multiple pairwise comparisons were conducted with Dunn’s test. Relationships between variables were analyzed based on Pearson’s correlation coefficient (for variables with normal distribution) or Spearman’s correlation coefficient (in the case of non-normally distributed variables). The analysis used the significance level *p* < 0.05.

### 2.4. Ethical Considerations

The local bioethics committee gave its authorization for the research. Before beginning the study, the respondents were informed about the anonymous and voluntary nature of the survey. The written consent to participate in the study was obtained from each respondent. The study was conducted under the recommendations of the Helsinki Declaration elaborated by the World Medical Association [21] and the guidelines of Good Clinical Practice [22]. The study was approved by the Local Bioethics Committee at Wrocław Medical University on 8 June 2017 (ethical approval code: KB-388/2017).

## 3. Results

### 3.1. Characteristics of the Study Group

The study involved 102 patients (30 women and 72 men). The mean age of the respondents was 61.65 ± 13.62 years. Most of the surveyed patients were city dwellers, which accounted for 68% (69/102). Higher education was accounted for almost 20% (20/102; 19.61%) of the respondents, 24.51% (25/102) of the respondents had secondary education, while about 14% (14/102; 13.73%) declared primary education. The highest percentage (43/102; 42.16%) reported vocational education. The majority of the respondents were working people (51/102; 50%), nearly 6% (6/102; 5.88%) were unemployed, while 44.12% (45/102) of respondents were pensioners. The epidemiological information about the prevalence of chronic diseases indicated the coexistence of hypertension (69/102; 67.65%), lipid disorders (30/102; 29.41%), diabetes (30/102; 28.43%), and asthma (9/102; 8.82%). The average time of hospitalization was 6.46 ± 3.14 days and ranged from 3 to 25 days. The rehabilitation time was 21.28 ± 0.81 days and ranged from 19 to 25 days. The percentage of patients who underwent myocardial infarction with ST-segment elevation was 61.76% (63/102). All patients (100%) underwent PCI treatment. Most respondents were non-smokers (83/102; 81.37%). The demographic and clinical characteristics of respondents are presented in Table 1.

### 3.2. Readiness for Hospital Discharge (RHD-MIS) 

The study indicated low readiness in 48/102 (47.06%) of patients (48/102; 47.06%), while 27/102 (27.45%) were characterized by the intermediate level and 26/102 (25.49%) exhibited high readiness for discharge. 

Correlation analysis showed that age negatively correlated with the general RHD-MIS score (*r* = −0.398; *p* < 0.001). The analysis also showed a positive correlation between the hospitalization length and readiness (*r* = −0.355; *p* < 0.001). The analysis of the differences using the Mann-Whitney test showed higher readiness for discharge in patients living in relationships (*p* = 0.02), living with a family (*p* = 0.005), living in a city (*p* = 0.027), professionally active (*p* < 0.001), without diabetes (*p* = 0.008) and asthma (*p* = 0.037), but suffering from lipid disorders (*p* = 0.025). Moreover, the Kruskal-Wallis test showed that the general RHD-MIS score was higher in respondents with higher education than in the group with primary and vocational education. Additionally, the readiness for discharge was higher for secondary education than vocational education (*p* < 0.001). The results are presented in Table 2.

Regarding the RHD-MIS subscales, we observed that over half of the respondents (49.02%; 50/102) expressed a high level of subjective knowledge about coronary artery disease; whereas 34.31% (35/102) had a low level of subjective knowledge, and 17 respondents (16.67%) expressed an intermediate level. According to the correlation analysis, the patients’ age negatively correlated with subjective knowledge (*r* = −0.232; *p* = 0.019). Mann-Whitney’s test showed that the subjective knowledge was higher in people living in a partner relationship (*p* < 0.001), in a group of people living with a family (*p* = 0.001), professionally active (*p* < 0.001), with asthma (*p* = 0.017) and in patients after STEMI (*p* = 0.021). The Kruskal-Wallis statistics showed that patients’ subjective knowledge was higher in the group with tertiary and secondary education than in the group of patients with vocational education (*p* = 0.012). The results are shown in Table 3. A high level of objective knowledge was found in more than half of the respondents (66/102; 64.71%), the intermediate level was found in 32.35% (33/102) respondents, and a low level characterized only 3 (2.94%) respondents.

The correlation analysis showed that age correlated negatively with the level of objective knowledge about coronary artery disease (*r* = −0.397; *p* < 0.001). The Mann-Whitney test showed that the objective knowledge of respondents was higher for people living in a relationship (*p* = 0.033), living with family (*p* = 0.031) and living in a city (*p* = 0.02), professionally active (*p* = 0.002), and after STEMI (*p* = 0.007). The Kruskal-Wallis analysis proved that objective knowledge in patients was higher for tertiary and secondary education than in the group with vocational education (*p* = 0.001). The results are presented in Table 4.

Over half of the respondents (60/102; 58.82%) had low expectations of educational activities, 29 respondents (28.43%) had an intermediate level of expectations, and 13 patients had high expectations (12.75%). The expectations were lower for vocational education (*p* = 0.003). A negative correlation between age and the level of expectations was observed (*r* = −0.36; *p* < 0.001). According to the Mann-Whitney difference test, higher expectations were observed in professionally active people (*p* < 0.001), patients with diabetes (*p* = 0.001), individuals with lipid disorders (*p* = 0.008), and patients with longer hospitalization times (*p* < 0.001). The results of the expectations are presented in Table 5.

### 3.3. Acceptance of Illness (AIS)

The study showed an intermediate level of acceptance of illness (27.31 ± 8.78 points), which translates into 3.42 points per question. The AIS score was higher in men, for respondents in relationships, and living with a family, with secondary education and professionally active (*p* < 0.05). Patients after myocardial infarction with coexisting diseases, such as lipid disorders and asthma, had higher acceptance of illness than patients without these diseases (*p* = 0.023, *p* = 0.006). Spearman’s correlation analysis showed the negative correlations between acceptance of illness and lengths of hospitalization and rehabilitation and age, respectively (*r* = −0.432; *p* = 0.002, *r* = −0.301; *p* = 0.003, *r* = −0.298; *p* < 0.001). The AIS factor positively correlated with readiness for discharge in patients with MI, *r* = 0.523, their subjective, *r* = 0.389, and objective knowledge, *r* = 0.468, and expectations, *r* = 0.387, (all values of significance, *p* < 0.001). Detailed data are presented in Table 6.

## 4. Discussion

The study investigated readiness for discharge in patients after myocardial infarction treated with percutaneous coronary intervention (PCI). We examined the effects of demographic and clinical variables on readiness for discharge and tested correlations between the AIS and the RHD-MIS measures. Our research showed that low readiness characterized nearly half of patients (47.06%), 27.45% of patients exhibited intermediate readiness, while 25.49% presented high readiness. As indicated by cross-sectional analysis, the AIS score may be the crucial factor contributing to increasing readiness to discharge in cardiac patients (*r* = 0.523).

The present research aimed primarily to recognize multiple factors that could affect the implementation of discharge planning, including patient readiness for discharge after myocardial infarction. To this end, this work attempted to investigate the relationship between socio-demographic and clinical variables and readiness for hospital discharge. The age factor turned out to be an essential variable, as younger patients with MI may exhibit higher readiness for discharge than older patients. This is consistent with studies among elderly patients with atrial fibrillation, frailty syndrome, who accept the disease to a lesser extent [23]. This study showed that correlations between the level of readiness to discharge in patients after myocardial infarction and the degree of the acceptance of illness were significant. However, in this area of research, there are no conclusive results. According to Kubica et al., there was also a higher level of health education among young people [24]. On the other hand, a study published by Briesacher et al. showed that people over 70 years of age represent a more remarkable ability to comply with the recommendations than the younger group [25]. 

Other variables affecting the readiness for discharge in patients after myocardial infarction are their marital and living status. In this study, the state of knowledge was significantly higher in people living in relationships and living with their families. The results can be explained by limited support from relatives in the therapeutic process. This finding is also supported by Mayberry et al., who noted that not receiving support from loved partners favors not following the therapeutic recommendations [26].

Studies have shown that the place of residence can also have a significant connection with the readiness for discharge [24,27]. Knowledge appropriate to the high level of readiness for discharge was demonstrated by respondents who declared to be city dwellers. Additionally, Kubica et al. noted that people living in cities showed significantly more knowledge and preparation [24]. Other reports, however, are presented in the work of Tsilimingras et al., where it was observed that there are no significant differences between respondents living in rural and urban areas [27].

Education has proved to be a factor that significantly correlates with readiness for discharge. The RHD-MIS general score was higher among patients with tertiary education than among people who received primary or vocational education. Moreover, respondents with secondary or tertiary education scored higher on the knowledge subscale than people with primary or vocational education. Other results by Kubica et al. showed that the respondents with secondary education obtained the highest level of knowledge [24]. In other studies, Tsilimingras et al. claim no connection between education and compliance with therapeutic recommendations [28].

Another variable that has proved statistically significant is professional activity. Readiness for discharge, and consequently, patient knowledge were higher in the group of professionally active patients than in the group of pensioners, who were characterized by significantly higher results than in the group of inactive people. Similar relationships between the patient’s knowledge and employment were demonstrated in the work of Kubica et al. [24].

The present study also assessed which of the socio-demographic and clinical variables significantly impacted respondents’ acceptance of illness. This psychological factor turned out that adaptation to the disease, i.e., the level of acceptance of illness, was significantly related to gender and age. The acceptance of illness was significantly higher in men. Moreover, older age was associated with a lower level of acceptance of illness. Other results were reported by Spatola et al., who demonstrated that females and people over 55 years of age had lower AIS scores [29].

In this study, we found that acceptance of illness was higher in the group with secondary education than in the group with primary and vocational education. Like Besen and Esen, people with secondary education had a higher level of acceptance of illness than people with primary education [30]. It also turned out that acceptance of illness may depend on patients’ professional activity. We found that non-working patients showed a lower level of acceptance of illness. According to Besen and Esen, acceptance of illness among type II diabetes patients does not depend on professional activity [30]. In our study, higher AIS scores were observed among people in relationships than single people. Similarly, marital status was associated with the illness acceptance rate in a study by Van Damme-Ostapowicz et al. [31].

This study showed that the hospitalization and rehabilitation lengths of stay were significantly related to acceptance of illness. It turned out that the more extended the stay in the medical unit, the lower the level of acceptance of illness. This is consistent with the study by Łuczyk et al. showing that frequent hospitalizations may negatively affect patients’ acceptance of illness [32]. 

### Limitations of the Study

The limitations of this work include a relatively small sample size of patients after myocardial infarction (*n* = 102), and the data originated from only one administrative region. The study was conducted in a single university hospital, and the same results may not reflect the patients with MI assigned to other cardiology departments. Additional studies should be carried out to assess the readiness for hospital discharge among patients after MI because this group, if adhering to treatment and lifestyle modification, have a favorable long-term prognosis. The clinical importance of demographic and clinical factors for assessment of readiness to discharge can be problematic to establish based on the *p*-values only. Further in-depth analysis should consider multiple logistic regression to precisely distinguish the clinical importance for each variable included in the study.

## 5. Conclusions

Patients with MI after hospitalization are especially vulnerable to adverse events including hospital readmissions, complications, medical errors, etc. The effective implementation of discharge planning for MI patients should include objective assessments of several factors, including readiness for hospital discharge and acceptance of illness. The factor of readiness for discharge in MI patients may be higher in young people, in relationships, living with their families, with tertiary or secondary education, and those who are professionally active. The study suggests that acceptance of illness may enhance readiness for hospital discharge after myocardial infarction. However, this finding was based on a cross-sectional design that limits inferences about causality between both variables. In addition, a higher AIS score may be observed in patients with tertiary or secondary education and professionally active, with a short period of hospitalization and rehabilitation. Our study suggests, therefore, that effective preparation of patients with MI for discharge depends on psychological and demographic factors.

## 6. Practice Implications

In clinical practice settings, the effectiveness of education programs and prevention of myocardial infarction depends on patient readiness for hospital discharge. To this end, it is recommended to use the objective assessment of readiness for hospital discharge in patients after myocardial infarction in everyday clinical practice. The proposed assessment enables the identification of patient knowledge deficits and to plan effective educational measures in order to increase adherence to therapeutic recommendations and reduce the risk of re-hospitalization for acute coronary syndromes (ACS) and the costs of health services. As a result, patient education based on objective measurements will improve compliance with long-term treatment recommendations.

## Figures and Tables

**Table 1:** Socio-demographic and clinical characteristics of myocardial infarction (MI) patients.

Characteristics	*n*	%
Gender		
Female	30	29.41
Male	72	70.59
Age (years)		
30–40	9	8.82
41–50	12	11.76
51–60	24	23.53
61–70	29	28.43
71–80	22	21.57
Marital status		
In relationship	67	65.69
Single	34	33.33
No answer	1	0.98
Residence		
With family	71	69.61
Alone	31	30.39
Education		
Primary	14	13.73
Vocational	43	42.16
Secondary	25	24.51
Tertiary	20	19.61
Professional activity		
Yes	51	50.00
No	6	5.88
Pensioner	45	44.12
Comorbidities		
Arterial hypertension	69	67.65 *
Diabetes	29	28.43 *
Lipid disorders	30	29.41 *
Kidney failure	2	1.96 *
Asthma	9	8.82 *
ACS		
STEMI	63	61.76
NSTEMI	30	29.41
No answer	9	8.82
Treatment		
PCI	102	100
Smoking		
Yes	18	17.65
No	83	81.37
No answer	1	0.98
Hospitalization (days)		
0–5	46	45.1
6–10	50	49.02
11–15	2	1.96
16–20	0	0.00
21–25	2	1.96
No answer	1	1.96
Rehabilitation (days)		
19	1	0.98
20	6	5.88
21	63	61.76
22	26	25.49
23	1	0.98
24	0	0.00
25	2	1.96
No data	3	2.94

ACS, acute coronary syndrome; STEMI, ST-segment elevation acute coronary syndrome; NSTEMI, non-ST-segment elevation acute coronary syndrome; PCI, percutaneous coronary intervention. * percentages do not sum up to 100, as this was a multiple-choice question.

**Table 2:** Effects of socio-demographic and clinical variables on general readiness for hospital discharge in MI patients.

General Readiness for Discharge						
Variable	Feature	*n* = 102	M ± SD	Med.	Q1–Q3	*p* ^1^
Gender	Women	30	45.27 ± 11.44	43	39.25–55.25	0.296
	Men	72	48.18 ± 11.85	45	40–58	
Marital status	In relationship	67	49.43 ± 11.8	50	40–60	0.02 *
	Single	34	43.44 ± 10.82	41.5	35.25–50.75	
Residence	With family	71	49.61 ± 11.79	50	40–60	0.005 *
	Alone	31	42.09 ± 10	40	34–48.5	
Place of residence	Village	33	43.6 ± 11.45	41	39–53	0.027 *
	City	69	49.1 ± 11.55	49	40–60	
Education	Primary	14	46.21 ± 10.33	41.5	40–55.25	<0.001 ^3^^,^*
	Vocational	43	42.05 ± 9.51	41	36–48	
	Secondary	25	51.19 ± 13.3	53	40–64	
	Tertiary	20	54.6 ± 9.93	55	51.25–63	
Professional activity	Yes	51	53.18 ± 11.28	56	41.5–63	0.001 ^3,^*
	No	6	30.77 ± 2.28	31.5	30.25–32	
	Pensioner	45	42.89 ± 8.63	41	39–49	
Arterial hypertension	Yes	69	46.71 ± 10.88	43	40–56	0.515
	No	33	48.59 ± 13.49	50	38–62	
Diabetes	Yes	29	41.98 ± 10.07	41	34–50	0.008 *
	No	73	49.44 ± 11.76	48	40–60	
Lipid disorders	Yes	30	51.35 ± 13.24	53	42.5–63	0.025 *
	No	72	45.64 ± 10.73	42.5	39–54.5	
Asthma	Yes	9	39.76 ± 7.23	40	39–41	0.037 *
	No	93	48.05 ± 11.87	47	40–58	
ACS	STEMI	63	48.37 ± 11.49	45	40–58	0.055
	NSTEMI	30	43.78 ± 12.21	41	35–51.75	
Smoking	Yes	18	47.14 ± 11.76	42.5	40–60	0.982
	No	83	47.32 ± 11.88	45	39.5–56	
					*r*	*p* ^2^
Age					−0.398	<0.001 *
Hospitalization length of stay					−0.355	<0.001 *
Rehabilitation length of stay					−0.173	0.087
**RHD-MIS**						
**Variable**		**Number of points**	**Interpretation**		***n*** **= 102**	**%**
Readiness for hospital discharge		0–43	Low level		48	47.06
		44–57	Intermediate level		28	27.45
		58–69	High level		26	25.49

^1^ Mann-Whitney test, ^2^ Spearman correlation coefficient, ^3^ Kruskal-Wallis test + post-hoc analysis (Dunn test), * statistically significant results (*p* < 0.05). SD, standard deviation; Med., median; Q, quartiles; RHD-MIS, Readiness for Hospital Discharge After Myocardial Infarction Scale.

**Table 3:** Effects of socio-demographic and clinical variables on subjective knowledge about the disease in MI patients.

RHD-MIS Subjective Knowledge						
Variable	Feature	*n*	M ± SD	Med.	Q1–Q3	*p* ^1^
Gender	Women	30	16.11 ± 3.58	16.5	12.7–19	0.068
	Men	72	17.05 ± 4.44	19	14.75–21	
Marital status	In relationship	67	18.01 ± 3.38	19	16–21	<0.001 *
	Single	34	14.37 ± 4.72	15	12–18	
Residence	With family	71	17.81 ± 3.39	19	16–21	<0.001 *
	Alone	31	14.4 ± 4.95	15	11.83–19	
Place of residence	Village	33	15.5 ± 5.19	17	12–20	0.122
	City	69	17.39 ± 3.53	19	15–20	
Education	Primary	14	14.29 ± 5.9	16	11.25–19	0.012 ^3,^*
	Vocational	43	16.03 ± 3.85	16	12.72–19.5	
	Secondary	25	17.92 ± 3.97	20	17–21	
	Tertiary	20	18.7 ± 2.49	19.5	18–20.25	
Professional activity	Yes	51	18.16 ± 3.4	19	17–21	<0.001 ^3,^*
	No	6	9.77 ± 5.41	11.8	5–13.65	
	Pensioner	45	16.14 ± 3.86	16	12–20	
Arterial hypertension	Yes	69	17.14 ± 3.77	19	14–21	0.328
	No	33	16.02 ± 4.98	18	13–20	
Diabetes	Yes	29	16.38 ± 4.05	17	12.83–21	0.569
	No	73	16.93 ± 4.29	19	15–20	
Lipid disorders	Yes	30	16.82 ± 3.84	17.5	13.25–20	0.833
	No	72	16.76 ± 4.38	18.5	13.75–20	
Asthma	Yes	9	13.76 ± 3.39	12	12–13	0.017 *
	No	93	17.07 ± 4.18	19	15–20	
ACS	STEMI	63	17.06 ± 4.51	19	15–20.5	0.021 *
	NSTEMI	30	15.34 ± 3.6	15	12–18.5	
Smoking	Yes	18	15.89 ± 6.01	19	13.5–19.75	0.7
	No	83	16.99 ± 3.75	18	13.5–20	
					*r*	*p* ^2^
Age					−0.232	0.019
Hospitalization length of stay					−0.012	0.905
Rehabilitation length of stay					−0.107	0.293
**RHDS MIS**						
**Variable**		**Number of points**	**Interpretation**		***n***	**%**
Readiness for hospital discharge		0–43	Low level		35	34.31
		44–57	Intermediate level		17	16.67
		58–69	High level		50	49.02

^1^ Mann-Whitney test, ^2^ Spearman correlation coefficient, ^3^ Kruskal-Wallis test + post-hoc analysis (Dunn test), * statistically significant results (*p* < 0.05). SD, standard deviation; Med. median; Q, quartiles.

**Table 4:** Effects of socio-demographic and clinical variables on objective knowledge about the disease in MI patients.

RHD-MIS Objective Knowledge						
Variables	Feature	*n*	M ± SD	Med.	Q1–Q3	*p* ^1^
Gender	Women	30	18.48 ± 2.13	19	17–20	0.33
	Men	72	18.68 ± 2.58	19.92	18–20.25	
Marital status	In relationship	67	19.03 ± 2.14	20	18–21	0.033 *
	Single	34	17.86 ± 2.86	19	17–20	
Residence	With family	71	18.96 ± 2.21	20	18–21	0.031 *
	Alone	31	17.84 ± 2.81	19	17–20	
Place of residence	Village	33	17.59 ± 3.05	18	16.33–20	0.02 *
	City	69	19.11 ± 1.94	19	18–21	
Education	Primary	14	17 ± 3.62	18	13–20	0.001 ^3,^*
	Vocational	43	17.98 ± 2.42	18	17–20	
	Secondary	25	19.39 ± 1.53	20	19–21	
	Tertiary	20	20.15 ± 0.99	20.5	19–21	
Professional activity	Yes	51	19.25 ± 2.34	20	19–21	0.002 ^3^
	No	6	17.83 ± 2.23	18	17.25–19.5	
	Pensioner	45	18.01 ± 2.46	19	17–20	
Arterial hypertension	Yes	69	18.44 ± 2.43	19	17–20	0.124
	No	33	19 ± 2.49	20	18–21	
Diabetes	Yes	29	18.39 ± 2.42	19	17–20	0.376
	No	73	18.71 ± 2.47	19	18–21	
Lipid disorders	Yes	30	19.04 ± 2.27	20	18–21	0.189
	No	72	18.44 ± 2.52	19	17–20	
Asthma	Yes	9	17.33 ± 3.35	18	17–19	0.181
	No	93	18.74 ± 2.33	19	18–20	
ACS	STEMI	63	18.92 ± 2.39	20	18–21	0.007 *
	NSTEMI	30	17.61 ± 2.59	17.5	17–19.75	
Smoking	Yes	18	18.5 ± 3.22	20	17.25–21	0.417
	No	83	18.65 ± 2.29	19	17.5–20	
					*r*	*p* ^2^
Age					−0.397	<0.001 *
Hospitalization length of stay					−0.171	0.089
Rehabilitation length of stay					−0.185	0.066
**RHDS MIS**						
**Variable**	**Number of points**		**Interpretation**		***n***	**%**
Objective knowledge	0–43		Low level		3	2.94
	44–57		Intermediate level		33	32.35
	58–69		High level		66	64.71

^1^ Mann-Whitney test, ^2^ Spearman correlation coefficient, ^3^ Kruskal-Wallis test + post-hoc analysis (Dunn test), * statistically significant results (*p* < 0.05). SD, standard deviation; Med., median; Q, quartiles.

**Table 5:** Effects of socio-demographic and clinical variables on expectations in MI patients.

RHD- MIS Expectations						
Variable	Feature	*n*	M ± SD	Med.	Q1–Q3	*p* ^1^
Gender	Women	30	10.69 ± 8.22	12	0.5–17	0.377
	Men	72	12.44 ± 9.29	12	3.5–19.25	
Marital status	In relationship	67	12.4 ± 9.23	12	2.5–20.29	0.608
	Single	34	11.21 ± 8.17	11.5	2–17.25	
Residence	With family	71	12.84 ± 9.43	12	4.5–20.79	0.15
	Alone	31	9.84 ± 7.58	9	2–17	
Place of residence	Village	33	10.52 ± 8.22	9	2–17	0.23
	City	69	12.6 ± 9.31	12	2–20.57	
Education	Primary	14	14.93 ± 6.07	17	9.75–18.5	0.003 ^3,^*
	Vocational	43	8.04 ± 7.31	9	0–13.5	
	Secondary	25	13.88 ± 11.09	12	1–26	
	Tertiary	20	15.75 ± 8.3	15	11.75–22	
Professional activity	Yes	51	15.78 ± 8.81	18	9.5–23	<0.001 ^3,^*
	No	6	3.17 ± 4.54	0.5	0–7	
	Pensioner	45	8.73 ± 7.51	9	1–15	
Arterial hypertension	Yes	69	11.14 ± 8.86	12	1–18	0.192
	No	33	13.58 ± 9.15	13	5–22	
Diabetes	Yes	29	7.21 ± 7.48	9	0–12	<0.001 *
	No	73	13.8 ± 8.88	14	7–21	
Lipid disorders	Yes	30	15.49 ± 9.29	18	9.75–22	0.008 *
	No	72	10.44 ± 8.48	9.5	1.75–17.25	
Asthma	Yes	9	8.67 ± 6.87	9	1–13	0.257
	No	93	12.24 ± 9.13	12	2–20	
ACS	STEMI	63	12.39 ± 8.75	12	6–19	0.404
	NSTEMI	30	10.83 ± 9.32	12	0–17	
Smoking	Yes	18	12.75 ± 8.45	12	8.25–20.43	0.665
	No	83	11.67 ± 9.16	12	1.5–18	
					*r*	*p* ^2^
Age					−0.36	<0.001 *
Hospitalization length of stay					−0.385	<0.001 *
Rehabilitation length of stay					−0.143	0.159
**RHDS MIS**						
**Variable**	**Number of points**		**Interpretation**		***n***	**%**
Expectations	0–43		Low level		60	58.82
	44–57		Intermediate level		29	28.43
	58–69		High level		13	12.75%

^1^ Mann-Whitney test, ^2^ Spearman correlation coefficient, ^3^ Kruskal-Wallis test + post-hoc analysis (Dunn test), * statistically significant results (*p* < 0.05). SD, standard deviation; Med., median; Q, quartile values.

**Table 6:** Effects of socio-demographic and clinical variables on acceptance of illness (AIS) in patients after myocardial infarction.

AIS						
Variable	Feature	*n*	M ± SD	Med.	Q1–Q3	*p* ^1^
AIS		102	27.31 ± 8.78	29.86	20 −35.75	
Gender	Women	30	23.21 ± 7.66	22.5	18.25–30	0.001 *
	Men	72	29.02 ± 8.7	31.5	20.75–37	
Marital status	In relationship	67	29.58 ± 8.46	32	23.5–36.5	<0.001 *
	Single	34	22.64 ± 7.64	21.5	18–29	
Residence	With family	71	29.69 ± 8.37	32	24.5–36.5	<0.001 *
	Alone	31	21.85 ± 7.19	21	18–25.07	
Place of residence	Village	33	25 ± 8.57	24	18–32	0.051
	City	69	28.42 ± 8.72	30	21–36	
Education	Primary	14	24.07 ± 7.4	22	18–30	
	Vocational	43	25.22 ± 8.3	25	19–32	
	Secondary	25	31.25 ± 8.86	36	24–38	0.001 ^3,^*
	Tertiary	20	29.15 ± 8.9	30.5	26.25–36.25	
Professional activity	Yes	51	31.59 ± 7.22	34	30–37	<0.001 ^3,^*
	No	6	19.17 ± 7.7	16	14.5–19.75	
	Pensioner	45	23.55 ± 8.14	23	18–29.71	
Arterial hypertension	Yes	69	26.42 ± 8.51	29	20–33	0.113
	No	33	29.17 ± 9.17	34	19–37	
Diabetes	Yes	29	26.13 ± 8.39	28	19–31	0.329
	No	73	27.78 ± 8.95	30	20–36	
Lipid disorders	Yes	30	30.46 ± 7.67	32	26.75–37	0.023 *
	No	72	26 ± 8.93	25.07	18–34	
Asthma	Yes	9	19.67 ± 7.92	18	18–28	0.006 *
	No	93	28.05 ± 8.54	30	21–36	
ACS	STEMI	63	26.74 ± 9.24	29.71	19.5–34.5	0.895
	NSTEMI	30	27.13 ± 8.43	29	18.25–35.5	
Smoking	Yes	18	28 ± 8.7	30	20–37.75	0.638
	No	83	27.05 ± 8.83	29	19–34.5	
					*r*	*p* ^2^
Age					−0.432	<0.001 *
Hospitalization length of stay					−0.301	0.002 *
Rehabilitation length of stay					−0.298	0.003 *
**RHD-MIS vs. AIS**						
General readiness for discharge					0.523	<0.001 *
Subjective knowledge					0.389	<0.001 *
Objective knowledge					0.468	<0.001 *
Expectations					0.387	<0.001 *

^1^ Mann-Whitney test, ^2^ Spearman correlation coefficient, ^3^ Kruskal-Wallis test + post-hoc analysis (Dunn test), * statistically significant results (*p* < 0.05). SD, standard deviation; Med., median; Q, quartiles.

## Data Availability

The data that support the findings of this study are available from the corresponding author, upon reasonable request.
